# Transcervical approach for carotid artery stenting with transitory reversal flow: Case report

**DOI:** 10.1016/j.ijscr.2021.106206

**Published:** 2021-07-17

**Authors:** E. Dinoto, F. Ferlito, G. Tortomasi, S. Evola, G. Bajardi, F. Pecoraro

**Affiliations:** a Vascular Surgery Unit, AOUP Policlinico ‘P. Giaccone’, Palermo, Italy; b Department of Surgical, Oncological and Oral Sciences, University of Palermo, Italy; c Unit of Cardiology, Department of Health Promotion, Mother and Child Care, Internal Medicine and Medical Specialties (ProMISE) ‘G. D'Alessandro’, University Hospital Paolo Giaccone, University of Palermo, Palermo, Italy

**Keywords:** Transcervical approach, Reversal flow, Carotid artery stenting, Case report

## Abstract

**Introduction:**

Carotid artery stenting (CAS) has been indicated as an alternative to carotid endarterectomy in high risk patients. Sometimes, an aortic arch can be anatomically unfavourable for CAS. Herein we report our experience in a case of CAS with transcervical approach.

**Presentation of case:**

A 77-year-old male was referred to our hospital for severe subtotal occlusion of the left internal carotid artery. He had a past medical history of radiation to the head and neck for laryngeal cancer. Previous CT-angiography had shown a type III aortic with bovine arch. CAS via transcervical approach was performed with transitory reversal flow during the placement of RX Spider Filter 6 Fr (Medtronic, Minneapolis, MN). After release of 7 × 30 mm RX Xact carotid stent (Abbott Vascular, Chicago, IL) and ballooning with a 5.5 × 30 mm Rx Submarine balloon catheter (Medtronic Minneapolis, MN), angiography check showed a good result.

**Discussion:**

The transcervical approach is an innovative technique where usually a shunt is created, either between the common carotid artery and the internal jugular vein or between the common carotid artery and the common femoral vein. This flow reversal reduces the risk of periprocedural embolic events. In our experience a short proximal clamping with transitory reversal flow, reduces the invasiveness of procedure with good outcomes.

**Conclusion:**

Transcervical carotid access with transitory reversal flow is a valid alternative in complicated patient with anatomy unfit for CAS.

## Highlights

• Carotid stenting can be an alternative to endarterectomy in high risk patients.
• Aortic arch can be anatomically unfavourable for Carotid artery stenting.
• Transcervical approach is a technique suited to patients unfit for stenting.
• Transcervical access with transitory reversal flow is useful in complicated patient.

## 1 Introduction

The benefit of carotid endarterectomy (CEA) for stroke prevention has been demonstrated in several randomized controlled trial in both symptomatic patients and asymptomatic patients with carotid stenosis [1], [2], [3]. Perioperative outcomes of CEA are related to patient risk factors, so carotid artery stenting (CAS) has been indicated as an alternative to CEA in high risk patients [4], [5]. However, sometimes, an aortic arch can be anatomically unfavourable for CAS.

In such cases, the transcervical approach may be preferred, especially, when femoral o radial access is not practicable. Herein we report our experience in a case of CAS with transcervical approach after an attempt with usual accesses.

This work has been written in accordance with the SCARE criteria [6].

## 2 Case report

**Fig. 1:**
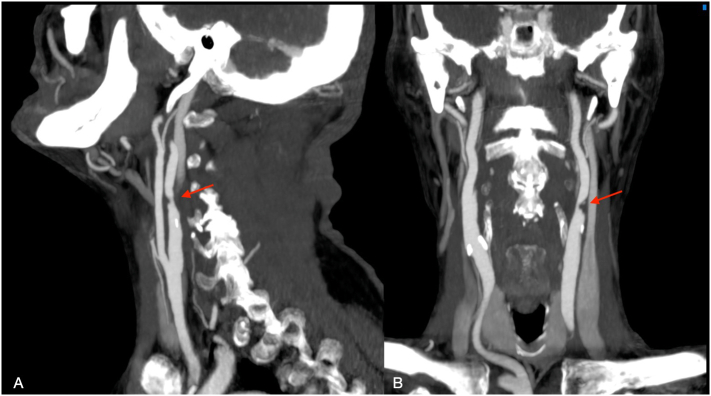
Preoperative CT Angiography showing stenosis of left internal carotid artery in sagittal plane (A) and coronal plane (B).

A 77-year-old male with hypertension, diabetes mellitus, was referred to our hospital for elective internal carotid artery stenting in asymptomatic severe subtotal occlusion of the left internal carotid artery (Fig. 1). He had a past medical history of radiation to the head and neck for laryngeal cancer. No coronary artery disease or cardiac arrhythmias were reported.

**Fig. 2:**
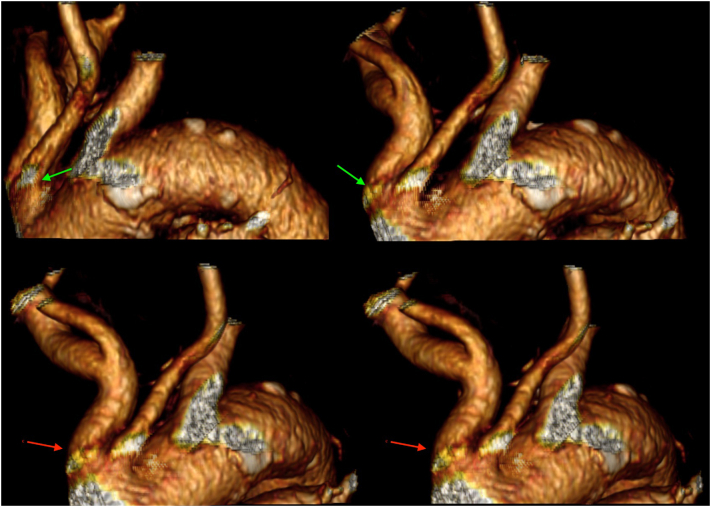
Preoperative CT Angiography 3-dimensional volume rendering showing aortic arch and left common Artery from different angles. The common origins of brachialcephalic artery and left common carotid are an important anatomical obstacle to the passage with access from femoral or radial access.

**Fig. 3:**
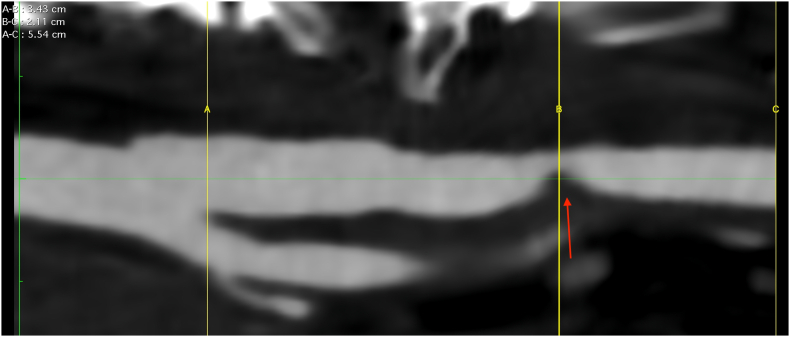
Preoperative CT Angiography centerline showing stenosis, distance from origin of internal carotid and length of plaque.

Previous CT-angiography had shown a type III aortic with bovine arch (Fig. 2). The patient was considered to be a poor candidate for carotid endarterectomy (CEA) due to a history of head and neck irradiation and a short neck, another distance of 3 cm from carotid bifurcation, made more difficult a surgical dissection (Fig. 3). Percutaneous carotid artery stenting (CAS) was initially attempted with access via the femoral and right radial approach. However, this procedure was unsuccessful due to a very tortuous and calcified type III aortic arch.

**Fig. 4:**
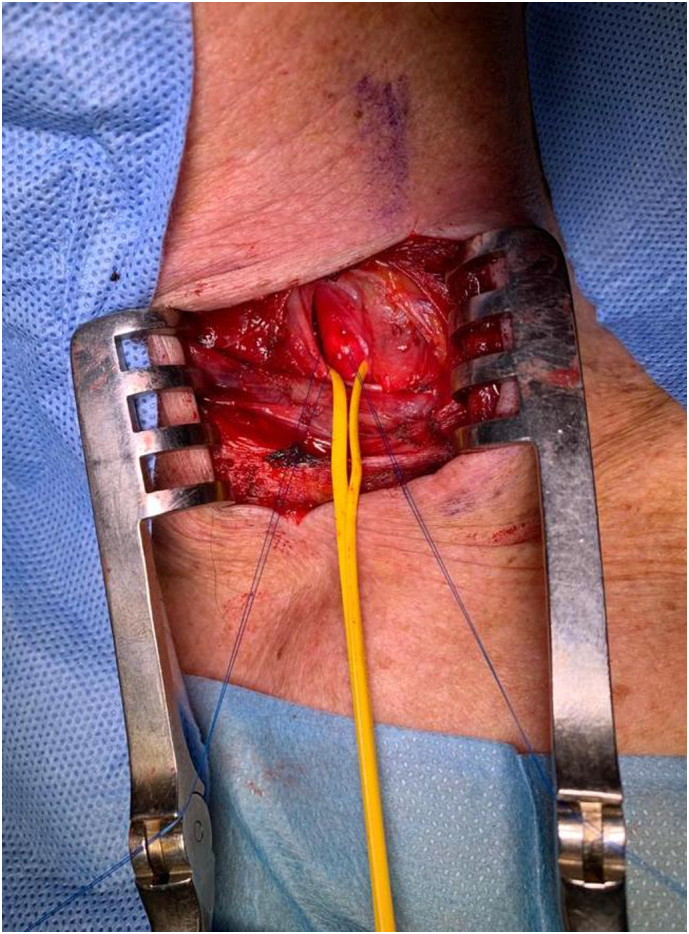
Surgical exposition of left common artery at the base of neck with surgiclose technique. After minimal surgical access to the common carotid artery, exposing only the anterior wall, 2 preliminary 5-0 polypropylene transmural single sutures were placed in the horizontal plane.

**Fig. 5:**
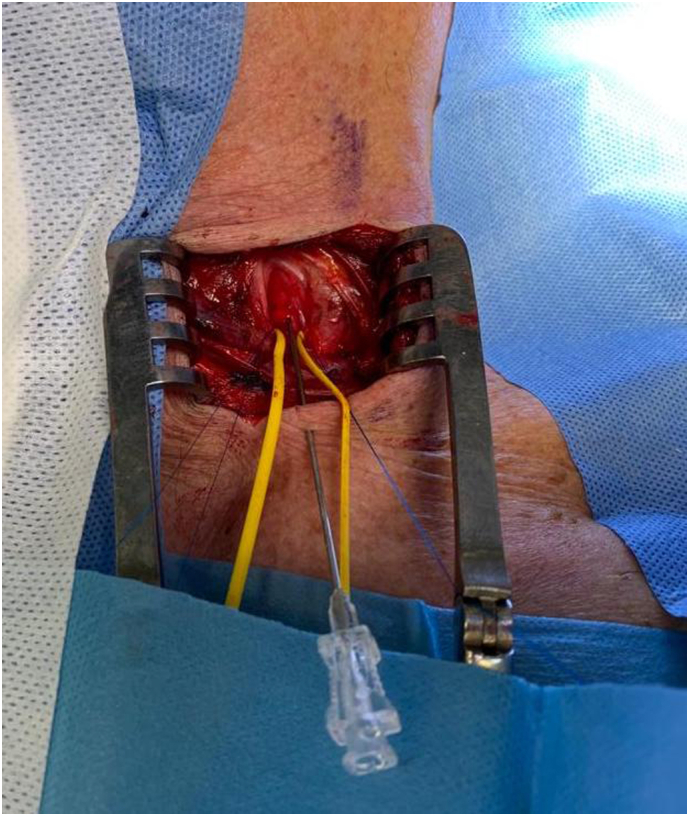
Puncture of left common artery through transcervical access. The vessel is accessed via an open Seldinger technique in the midline between the 2 sutures, and the sheath was then inserted over the wire.

**Fig. 6:**
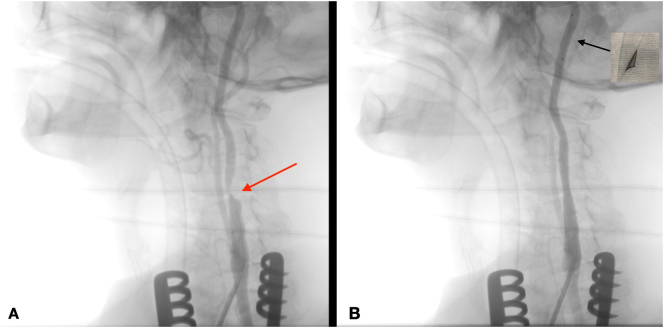
Intraoperative angiography showing carotid lesion before (A) and after Stenting with embolic protection device deployed inside the ipsilateral internal carotid (B).

**Fig. 7:**
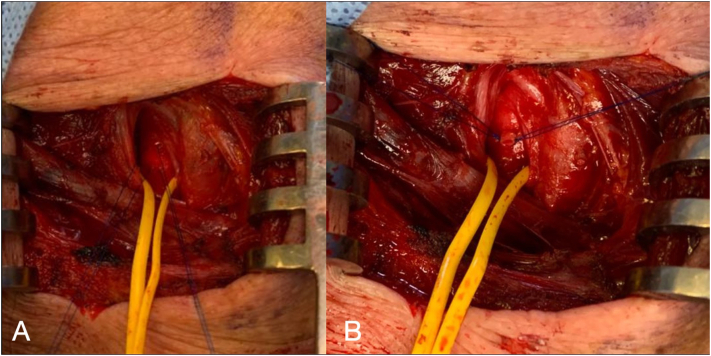
Surgiclose technique before (A) and after closure (B). At the end of the procedure, the sheath and wire were removed, and with digital pressure on the vessel distally, the access site is washed out in antegrade fashion. All 2 sutures are then pulled tight and tied.

**Fig. 8:**
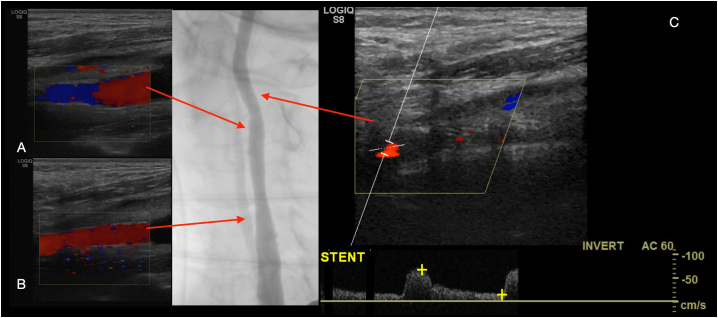
Ultrasound doppler showing stent in distal end (A), medium tract (B), blood flow after stent (C).

Carotid artery stenting (CAS) via the transcervical approach was then performed. The patient was given general anesthesia with the head turned towards the right side. The common carotid artery was exposed using surgical cutdown and exploration at the base of neck (Fig. 4). A sheath was introduced into the common carotid artery with passage through the skin of the incision proximally to allow for improved stability of the sheath (Fig. 5). To avoid a clamping post stenting, a gate access on common carotid was prepared with the surgiclose technique, an easy and fast trick, applicable to all vessels to avoid a damage of artery [7], [8]. The area of stenosis was identified with a carotid angiogram and, previous clamping of common carotid having a flow reversal, was crossed with a filter wire where a RX Spider Filter 6 Fr (Medtronic, Minneapolis, MN) was used distally in the left internal carotid artery, re-opening right after the artery. The lesion was treated with release of a 7 × 30 mm RX Xact carotid stent (Abbott Vascular, Chicago, IL). Post-dilation was then performed with a 5.5 × 30 mm Rx Submarine balloon catheter (Medtronic Minneapolis, MN), with good results (Fig. 6). The filter was retrieved followed by sheath removal. The arteriotomy site was easier closed thanks to surgiclose technique and hemostasis was successfully achieved (Fig. 7). The patient tolerated the procedure well and was successfully extubated. He was neurologically intact with National Institutes of Health Stroke Scale score of zero. Following the procedure, he remained on dual antiplatelet and statin therapy. At 3-months followup, Ultrasound doppler showed lack of restenosis with good flow intrastent (Fig. 8).

## 3 Discussion

Although CEA is currently considered as the gold standard treatment for patient with severe carotid stenosis, the use of the carotid artery stent (CAS) has become the preferred method for the management of patients considered to be at high risk for surgery. CAS is preferable to CEA in patients with severe cardiac and/or pulmonary comorbidity, and in those presenting with specific conditions, such as paralysis of the contralateral laryngeal nerve, stenosis extended to the cranial region or the clavicular region, restenosis, previous tracheostomy or surgical intervention/radiotherapy of the neck [9]. Patients who may have a poor outcome following CAS include patients of advanced age, patients with severe cardiopulmonary dysfunction, patients with advanced renal disease, and patients with anatomical changes that may challenge surgical access, or with prior irradiation to the neck [4], [5], [6], [7], [8], [9], [10], [11]. Conventional stenting procedures used for the carotid artery can be performed using various approaches for access. Most commonly, percutaneous access is achieved through the common femoral arteries, less frequently radial access. Angiography of the supra-aortic trunks and both extra and intracranial carotid and vertebral arteries was performed to determine the choice of catheters. Imaging evidence of significant carotid artery calcification or atherosclerotic disease increase risk of athero-embolization during the procedure [12], [13]. It is also more difficult to maneuver wires and catheters into an anatomically challenging arch, such as a type III arch, or when there is severe aortic tortuosity. Several studies have shown increased fluoroscopy time and increased complications in patients who have a type III aortic arch [14]. Transcervical approach reduces the risk of athero-embolization and improves postoperative outcome. In our case, the patient had an anatomically challenging type III aortic arch and a tortuosity in the origin of left common carotid (bovine arch) that made difficult and potentially dangerous to maneuver wires and catheters in aortic arch. Another, patient had a history of previous neck irradiation and had a lesion distant from carotid biforcation rendering surgical access for carotid endarterectomy (CEA) inappropriate. Therefore, a transcervical approach using a CAS was the best option for patient. In addition, surgiclose technique permitted to avoid a stenosis on common carotid artery [15], [16].

Standard CAS technique includes the use of a distal embolic filter passing through the stenosis while blood flow remains antegrade. In this case reported, a short carotid clamping was used to place a distal embolic filter with flow reversal so as to avoid embolism during the transition.

There are currently two embolic protection devices (EPDs) available for use: distal-EPDs and proximal-EPDs. RX Spider Filter 6 Fr (Medtronic, Minneapolis, MN), as was used in this case, is a type of distal-EPD. Although d-EPDs are more commonly used in clinical practice, current data support that p-EPDs more efficiently reduce the embolic risk than d-EPDs. Therefore, p-EPDs are more favorable in high-risk plaques (recently symptomatic and vulnerable plaque such as ulcerated, heterogeneous, high-lipid burden and the presence of intraplaque hemorrhage or intraluminal thrombus) [17]. The most commonly percutaneous pEPD used is the Mo.Ma device. This device system saves the cerebrum from embolic debris by two atraumatic balloons. One of these blocks the antegrade blood flow from the CCA, and the other blocks the retrograde blood flow from the ECA [18]. An alternative to transfemoral or transradial carotid is the transcervical approach where usually a shunt is created, either between the common carotid artery and the internal jugular vein or between the common carotid artery and the common femoral vein [19]. The common carotid artery is usually clamped proximally, and there is flow reversal from the common carotid artery into the venous system. The flow reversal system involves a filter that collects debris before returning the blood to the vein. This flow reversal reduces the risk of periprocedural embolic events [20]. CAS through a transcervical approach is a safe procedure with a very low incidence of stroke and complications. Two techniques are described in the literature: direct CAS with transcervical access and transcervical CAS under reversed flow [21]. In our experience a short proximal clamping with transitory reversal flow, permitted to exploit the advantages of transcervical approach and distal embolic protection device, reducing the invasiveness.

## 4 Conclusions

Transcervical carotid access with the use of a carotid artery stent (CAS) may be necessary for patients who are unfit for carotid endarterectomy (CEA). Typically, the method involves flow reversal of the carotid artery into a venous system. In this case presented, the method for the CAS procedure differed in that the reversal flow was transitory and limited to placement of a distal embolic filter, which is more commonly associated with the percutaneous femoral access approach. Further studies are needed to test the efficacy and risks associated with this technique.

## Ethical approval

None.

## Funding

None.

## Author contribution

Ettore Dinoto: study concept, design, data collection, data analysis, interpretation, writing the paper, final approval of the version to be submitted, **guarantor**.

Francesca Ferlito: study concept, design, data collection, data analysis, interpretation, final approval of the version to be submitted.

Graziella Tortomasi: study concept, design, data collection, final approval of the version to be submitted.

Salvatore Evola: study concept, design, data collection, final approval of the version to be submitted.

Guido Bajardi: study concept, design, data collection, data analysis, interpretation, final approval of the version to be submitted.

Felice Pecoraro: study concept, design, data collection, data analysis, interpretation, writing the paper, final approval of the version to be submitted.

## Guarantor

Ettore Dinoto.

## Consent

Written informed consent was obtained from the patient for publication of this case report and accompanying images. A copy of the written consent is available for review by the Editor-in-Chief of this journal on request.

## Provenance and peer review

Not commissioned, externally peer-reviewed.

## Declaration of competing interest

The authors have no ethical conflicts to disclose.

## References

[1] Peluso A., Turchino D., Petrone A., Giribono A.M., Bracale R., Del Guercio L., Bracale U.M. (2019). Standard carotid endarterectomy versus carotid artery stenting with closed-cell stent design and distal embolic protection: does the age matter?. Transl. Med. UniSa.

[2] Pecoraro F., Dinoto E., Mirabella D., Corte G., Bracale U.M., Bajardi G. (2012 Oct). Basal cerebral computed tomography as diagnostic tool to improve patient selection in asymptomatic carotid artery stenosis. Angiology.

[3] Rajamani K., Chaturvedi S. (2007 Nov). Surgery insight: carotid endarterectomy–which patients to treat and when?. Nat Clin Pract Cardiovasc Med..

[4] Bracale U.M., Del Guercio L., Machì P., Dinoto E., La Marca M.G., Pecoraro F., Porcellini M., Bajardi G., Bracale G. (2012). Carotid endarterectomy versus stenting in patients with contralateral carotid artery occlusion. J. Vasc. Endovasc. Surg..

[5] Reed A.B., Gaccione P., Belkin M., Donaldson M.C., Mannick J.A., Whittemore A.D., Conte M.S. (2003 Jun). Preoperative risk factors for carotid endarterectomy: defining the patient at high risk. J. Vasc. Surg..

[6] Agha R.A., Franchi T., Sohrabi C., Mathew G., for the SCARE Group (2020). The SCARE 2020 guideline: updating consensus Surgical CAse REport (SCARE) guidelines. Int. J. Surg..

[7] Mayer D., Rancic Z., Wilhelm M., Genoni M., Veith F.J., Lachat M. (2008 Jun). Improved hybrid technique for vascular access and closure. J. Endovasc. Ther..

[8] Dinoto E., Ferlito F., Urso F., Pakeliani D., Bajardi G., Pecoraro F. (2021 Jun). Mechanical rotational thrombectomy in long femoropopliteal artery and stent occlusion in COVID-19 patient: case report. Int. J. Surg. Case Rep..

[9] Lanza G., Setacci C., Cremonesi A., Ricci S., Inzitari D., de Donato G., Castelli P., Pratesi C., Peinetti F., Lanza J., Zaninelli A., Gensini G.F. (2014). Carotid artery stenting: second consensus document of the ICCS/ISO-SPREAD joint committee. Cerebrovasc. Dis..

[10] Dorresteijn L.D., Vogels O.J., de Leeuw F.E., Vos J.A., Christiaans M.H., Ackerstaff R.G., Kappelle A.C. (2010 Aug 1). Outcome of carotid artery stenting for radiation-induced stenosis. Int. J. Radiat. Oncol. Biol. Phys..

[11] Pecoraro F., Dinoto E., Pakeliani D., Ferlito F., Mirabella D., Lachat M., Farina A., Bajardi G. (2020 Jul). Endovascular treatment of spontaneous internal carotid artery dissection with proximal embolic protection device. Ann. Vasc. Surg..

[12] Assaad M., Berry A., Zughaib M. (2019 Jan). Transcervical carotid artery stenting without flow reversal: a report of two cases. Am J Case Rep..

[13] Dinoto E., Pecoraro F., Farina A., Viscardi A., Bajardi G. (2020). Simultaneous endovascular treatment of synchronous symptomatic acute type B aortic dissection and large infrarenal aortic aneurysm. Technical tips and case report. Int. J. Surg. Case Rep..

[14] Kasirajan K., Schneider P.A., Kent K.C. (2003). Filter devices for cerebral protection during carotid angioplasty and stenting. J. Endovasc. Ther..

[15] Mayer D., Rancic Z., Wilhelm M., Genoni M., Veith F.J., Lachat M. (2008 Jun). Improved hybrid technique for vascular access and closure. J. Endovasc. Ther..

[16] Pecoraro F., Corte G., Dinoto E., Badalamenti G., Bruno S., Bajardi G. (2016). Cinical outcomes of Endurant II stent-graft for infrarenal aortic aneurysm repair: comparison of on-label versus off-label use. Diagn. Interv. Radiol..

[17] Giordan E., Lanzino G. (2017 Oct 18). Carotid angioplasty and stenting and embolic protection. Curr. Cardiol. Rep..

[18] Gungoren F., Besli F., Tanriverdi Z., Kocaturk O., Tascanov M.B. (2019). Unusual complication of carotid artery stenting as the result of a proximal emboli protection device (the Mo.Ma): Iatrogenic common carotid artery dissection. Anatol. J. Cardiol..

[19] Criado E., Doblas M., Fontcuberta J., Orgaz A., Flores A. (2004 Mar). Transcervical carotid artery angioplasty and stenting with carotid flow reversal: surgical technique. Ann. Vasc. Surg..

[20] Sultan S., Elkady R., Barrett N., Hynes N. (2019 Jun 25). Endovascular management of saccular extracranial internal carotid artery aneurysm using transcervical carotid approach and flow reversal. J Vasc Surg Cases Innov Tech..

[21] Sfyroeras G.S., Moulakakis K.G., Markatis F., Antonopoulos C.N., Antoniou G.A., Kakisis J.D., Brountzos E.N., Liapis C.D. (2013 Nov). Results of carotid artery stenting with transcervical access. J. Vasc. Surg..

